# Study of Adsorption and Desorption Performances of Zr-Based Metal–Organic Frameworks Using Paper Spray Mass Spectrometry

**DOI:** 10.3390/ma10070769

**Published:** 2017-07-08

**Authors:** Xiaoting Wang, Ying Chen, Yajun Zheng, Zhiping Zhang

**Affiliations:** 1 School of Chemistry and Chemical Engineering, Xi’an Shiyou University, Xi’an 710065, China; 18629343797@163.com (X.W.); returnshiyou@xsyu.edu.cn (Y.Z.); 2 Clinical Analysis Laboratory, Xi’an Mental Health Center, Xi’an 710061, China; 13359287846@163.com

**Keywords:** adsorption and desorption, Zr-based material, paper spray mass spectrometry

## Abstract

The dynamic pore systems and high surface areas of flexible metal–organic framework materials make them excellent candidates to be used in different kinds of adsorption processes. However, the adsorption and desorption behaviors of therapeutic drugs on metal–organic frameworks in solution are not fully developed. Here, we systematically investigated the adsorption and desorption behaviors of a typical therapeutic drug, verapamil, over several Zr-based metal–organic frameworks [e.g., Zr-FUM, UiO-66(Zr), UiO-66(Zr)-NH_2_ and UiO-66(Zr)-2COOH] as well as ZrO_2_ in an acetonitrile solution by using paper spray mass spectrometry. In contrast to other materials, UiO-66(Zr)-2COOH demonstrated a superior adsorption performance to verapamil due to their strong acid-base and/or hydrogen-bond interactions, and the adsorption process fitted well with the pseudo-second-order kinetic model. As verapamil-adsorbed materials were used for desorption experiments, ZrO_2_ demonstrated the most favorable desorption performance, whereas UiO-66(Zr)-2COOH yielded the poorest desorption capability. These Zr-based materials had also been coated at the surface with filter papers for the analysis of various drugs and proteins in the process of paper spray mass spectrometry. The results demonstrated that among the studied materials, ZrO_2_-coated paper gave the most favorable desorption performance as a pure drug solution, whereas the paper from UiO-66(Zr) demonstrated the optimal capability in the analyses of therapeutic drugs in a complex matrix (e.g., blood) and a protein (e.g., myoglobin).

## 1. Introduction

Metal–organic frameworks (MOFs) [1,2] have received considerable attention over the past decades and have been applied in diverse fields including gas storage [3,4], clean energy [5,6,7], purification [8,9], adsorption and separation [10,11,12,13,14,15,16], catalysis [17,18,19], sensors [20], supercapacitors [21] and drug carriers [22,23,24], owing to their large pore sizes, high apparent surface areas and extraordinary degree of variability for both the organic and inorganic components in their structures. When MOFs are used as absorbents for purification, separation, catalysis, waste streams, liquid fuels, biomedicine and chromatography, their adsorption and desorption performances play significant roles in determining the final results. Therefore, investigating the adsorption and desorption is expected to help us understand the capacities of employed MOFs in a comprehensive way.

To date, numerous important advances have been made in the investigation of the adsorption and desorption properties of various compounds on MOFs, but those studies are mainly focused on volatile compounds including H_2_ [25,26], CO and CO_2_ [27,28,29,30], NO [31], H_2_O [32], benzene [33], toluene [34], 1,2-dichloroethane [35], and methanol [36]. Various experimental techniques such as gravimetric measurements [25], inelastic neutron scattering [37], infrared spectroscopy [38], thermal desorption spectroscopy [39], neutron diffraction [40] and electron paramagnetic resonance spectroscopy [41] have been intensively applied in the related investigation. These demonstrated that for MOFs, free binding sites at metal ions played a significant role for the adsorption properties of volatile compounds [4,42]. In addition, some attempts [43,44,45] have been made to study the adsorption and desorption of non-volatile compounds on MOFs in a solution. For example, Samokhvalov [45] comprehensively reviewed the recent development of adsorption and desorption of aromatic and heterocyclic compounds, including dyestuffs, agricultural chemicals, medicinal drugs, food additives and so on, on mesoporous MOFs in a solution. Stassin et al. [43] reported that UiO-66(Zr) was an effective adsorbent for the separation of lactic acid from aqueous (buffer) solutions based on hydrogen-bonding interactions with functional groups inside the pores, and due to the catalytic activity of UiO-66(Zr), desorption of the adsorbed lactic acid was performed in alcohols to recover up to 73% as ester, analyzed by a chromatography technique. Jun et al. [44] observed that MIL-100-Fe exhibited a much higher adsorption capacity for organoarsenic compounds (e.g., *p*-arsanilic acid and roxarsone) than activated carbon, zeolite, goethite and other MOFs via ultraviolet spectroscopy, and it could be facilely recycled by acidic ethanol.

Mass spectrometry (MS) has been demonstrated to be a powerful tool for chemical and biological analysis because of its high specificity, sensitivity and precision. For the purpose of improving the analysis sensitivity of various analytes, a variety of MOFs, including MIL-100(Fe) [46,47], MIL-101(Cr) [48], Zn_2_(bim)_4_ [49], UiO-66-NH_2_ [50], UiO-66-(OH)_2_ [51], have been applied as substrates for the ionization sources of MS. Lu et al. [52] summarized the applications of different types of MOFs in the analysis of small biological molecules, environmental pollutants and other low-molecular weight compounds. However, the above applications of MOFs are mainly limited in the matrix-assisted laser desorption/ionization (MALDI) coupling with time-of-flight mass spectrometry. To expand the application of MOFs in a mass spectrometric analysis, more recently we explored the capabilities of three types of MOFs [e.g., MIL-53(Al), ZIF-8 and UiO-66(Zr)] as substrates for paper spray MS, in which analyte transport was achieved by wicking a porous paper substrate into a high electric field [53], by coating them on the surfaces of filter paper. Among the studied materials, UiO-66(Zr) exhibited a superior performance in an analysis of therapeutic drugs in dried blood spots compared to other materials, thanks to its weak adsorption ability to tested drugs [54]. To further study the potential of Zr-based MOFs for a high sensitivity analysis of various analytes in paper spray MS, we systematically compared the adsorption and desorption capabilities of verapamil (see Figure S1 in the Supplementary Material) over ZrO_2_ and four types of Zr-based materials such as Zr-FUM, UiO-66(Zr), UiO-66(Zr)-NH_2_ and UiO-66(Zr)-2COOH in acetonitrile solution. Also, the performances of different MOFs with coated paper substrates have been compared in the elution or desorption of different therapeutic drugs and protein samples. 

## 2. Experimental Section

### 2.1. Chemicals and Materials 

The irregular ZrO_2_ particles with dimeters of approximately 1.0 μm were from Shanghai ST-Nano Science & Technology Co. Ltd. (Shanghai, China), and MOFs including Zr-FUM, UiO-66(Zr), UiO-66(Zr)-NH_2_ and UiO-66(Zr)-2COOH (Table 1 and Figure S2) with average diameters in the range of 1.0–5.4 μm were from Beijing HWRK Chem. Co. Ltd. (Beijing, China). The quantitative filter paper used for coating was from Hangzhou Special Paper Co. (Fuyang, China). The corn starch used as an adhesive agent was ordered from Jilin Zhiyou Technology Co., Ltd (Changchun, China). Therapeutic drug standards including amitriptyline, amisulpride, quetiapine, risperidone and verapamil were from Sigma-Aldrich (St. Louis, MO, USA). Myoglobin was ordered from Beijing Biodes Biotechnology Co. Ltd. (Beijing, China).

### 2.2. Preparation of Zro_2_- and MOF-Coated Paper Substrates 

The detailed procedure was similar to our recent studies [55,56]. Typically, 0.5 g ZrO_2_ or MOFs were dispersed into 100 mL of deionized water containing 0.1 g corn starch as an adhesive agent. For the purpose of obtaining a uniform suspension, the mixture was sonicated for 20 min. Then, the obtained suspension was directly transferred to a Buchner funnel covered by a piece of blank filter paper of 11 cm in diameter for coating. When the aqueous solution in the above suspension was completely penetrated through the filter paper, approximately 20 mL of absolute ethanol was applied for washing in order to get rid of the remaining water at the surface of the coated papers. The papers were then hung in a hood to dry for 2 h and were pressed between glass plates overnight for use. It should be pointed out that using the above method, ZrO_2_ and various MOFs could be uniformly coated at the surfaces of filter papers, and the coating thickness was in the range of 0.05–0.22 mm. 

### 2.3. Characterization of Zr-Based Materials and Coated Paper Substrates 

The crystal structures of Zr-based materials were characterized by X-ray diffraction (XRD) on an XRD-6000 diffractometer (Shimadzu Co., Chiyoda-ku, Tokyo, Japan) using Cu K_α_ radiation. FT-IR spectra of the obtained samples were recorded with a Thermo Nicolet 5700 Series infrared spectrometer (Thermo Fisher Scientific, Waltham, MA, USA) in transmission mode in the range of 4000–400 cm^−1^. The resolution was 4 cm^−1^ and 32 scans were signal-averaged in each interferogram. The N_2_ adsorption–desorption isotherm was determined on a Micromeritics ASAP 2020HD88 (Micromeritics, Co., Norcross, GA, USA) apparatus at the temperature of liquid nitrogen. The surface area was calculated by the Brunauer–Emmett–Teller method. The surface structures of as-prepared ZrO_2_- or MOF-coated papers were examined by using a JEOL JSM-6390A scanning electron microscope (SEM) (JEOL Ltd., Akishima, Tokyo, Japan). 

### 2.4. Adsorption Performance of Verapamil on Zro_2_ or Different MOFs 

The experiments were carried out by adding 0.4 g ZrO_2_ or different MOFs into 10 mL of acetonitrile solution containing 1 μg mL^−1^ verapamil, followed by stirring, respectively, under room temperature. At different adsorption times (e.g., 0, 0.25, 0.5, 1, 2.5, 5, 10, 20, 30, 45, 60, 90 and 120 min), 0.2 mL of the mixture was collected and centrifugated. Afterwards, 100 μL of the upper clear solution was pipetted, which was then diluted with 900 μL of acetonitrile for subsequent analysis. The remaining content of verapamil in the solution was analyzed with paper spray through the peak intensity of the fragment ion *m*/*z* 303 from verapamil. The paper substrate used was a quantitative filter paper, and the applied amount of sample solution was 25 μL.

The adsorption capacity of verapamil on different MOFs is calculated by *q*_t_ = (*C*_0_ − *C*_t_) *V*/*m*, in which *q*_t_ is the adsorption capacity, *C*_0_ and *C*_t_ are the concentrations of verapamil (mg L^−1^) before and after adsorption, *V* is the volume of solution (L), and *m* is the mass of the used ZrO_2_ or MOFs (g).

### 2.5. Desorption Performance of Verapamil from Verapamil-Adsorbed ZrO_2_ or MOFs

The experiments were performed by adding 50 μL 10 μg mL^−1^ verapamil solution (methanol as the solvent) into 0.4 g Zr-based materials, respectively, followed by stirring and air drying. Then, 10 mL of acetonitrile solution was added into the verapamil-adsorbed materials for desorption experiments. After continued stirring for fixed intervals (e.g., 0, 0.25, 0.5, 1, 2.5, 5, 10, 20, 30, 45, 60, 90 and 120 min), 0.2 mL of the mixture was collected and separated. The other procedures were the same as the above adsorption experiments.

### 2.6. Elution Behavior of Verapamil from Zro_2_- or MOF-Coated Papers 

Prior to the experiments, 2 μL of a 1:1 methanol/water solution or a blood sample containing verapamil (1 μg mL^−1^) was placed onto the prepared ZrO_2_- or MOF-coated paper substrates and the substrates were completely dried. Then, the papers were cut into triangles. The voltage was applied, and the solvent was applied multiple times to produce many paper spray events using the same substrate, bearing a single sample spot, while the signal of fragment ion *m*/*z* 303 from verapamil was monitored. Spray solvent of 25 μL was used each time, and the solvent was not added until the monitored ion signal decreased to a minimum, when the spray solvent was also exhausted. 

### 2.7. Paper Spray Analysis 

All experiments that involved paper spray were carried out with a commercial TSQ Quantum Access Max mass spectrometer (Thermo Fisher Scientific, San Jose, CA, USA) equipped with a homemade paper spray source. Mass spectra were recorded in a positive ion mode with a capillary temperature of 270 °C. The identification of analyte ions was confirmed by tandem mass spectrometry (MS/MS) using collision-induced dissociation (CID). Argon gas (99.995% purity) was used as a collision gas. The selected reaction monitoring (SRM) and instrumental parameters used for the therapeutic drug verapamil are as follows: verapamil, *m*/*z* 455→303; tube lens, 96 V; collision energy, 25 V.

To compare different protein charge state distributions, the average charge state (*Z_a_*_ve_) was used. This parameter is calculated using the following equation:

$$z_{ave}=\frac{\sum {}_{i}^{N}z_{i}W_{i}}{\sum {}_{i}^{N}W_{i}}$$

where *N* is the total number of protein charge states observed in the mass spectrum and *W_i_* is the signal intensity of the *z_i_* charge state [57].

## 3. Results and Discussion

### 3.1. Comparison of Adsorption and Desorption Capabilities in Acetonitrile Solution 

**Figure 1:**
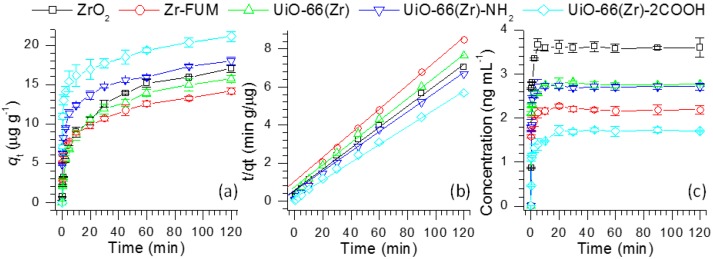
(**a**) Time profiles of verapamil adsorption over various Zr-based materials and (**b**) their derived pseudo-second-order kinetics plots of verapamil adsorption, and (**c**) time profiles of verapamil desorption from verapamil-adsorbed Zr-based materials.

As displayed in Figure 1a, the adsorbed quantities of verapamil over UiO-66(Zr)-2COOH are much higher than those using other Zr-based materials at all adsorption times. The relatively high adsorption capacity of UiO-66(Zr)-2COOH is likely not a result of its high porosity because the surface areas and pore volumes of Zr-FUM, UiO-66(Zr) and UiO-66(Zr)-NH_2_ are much higher than those of UiO-66(Zr)-2COOH (Table 1). In addition, although the surface areas of different MOFs are extremely higher than that of ZrO_2_, the adsorption feature of the less porous ZrO_2_ is comparable to some, such as UiO-66(Zr) and UiO-66(Zr)-NH_2_ (Figure 1a). To our knowledge, this case could be attributed to their various surface properties (e.g., acid-base properties), thus leading to different interactions between verapamil and those materials. For example, with a variation of the functional groups at the surfaces of UiO-66(Zr), UiO-66(Zr)-NH_2_ and UiO-66(Zr)-2COOH, their adsorption performances demonstrate an increasing trend, probably resulting from the more favorable formation of acid-base or/and hydrogen-bond interactions between UiO-66(Zr)-2COOH and verapamil than those from UiO-66(Zr) and UiO-66(Zr)-NH_2_. 

To gain an insight into their adsorption rate, the adsorption kinetic behaviors of verapamil on Zr-based materials were investigated using the pseudo-first-order and pseudo-second-order kinetic models. Table 2 lists the obtained adsorption kinetic parameters. Obviously, when the pseudo-first-order kinetic model was used, the experimental data (*q*_e_) are much higher than the calculated values, which could be due to the low correlation coefficients R^2^ in the range of 0.9301–0.9724. However, as the pseudo-second-order kinetic model is employed, the calculated values remain close to the experimental data. The correlation coefficients R^2^ are all higher than 0.9920 (Figure 1b), suggesting that the adsorption process of verapamil on the Zr-based materials fits well with the pseudo-second-order kinetic model.

The adsorption process plays an important role in reducing target compounds in solution or gas phases, whereas the desorption of compounds from an adsorbent is commonly accomplished in organic solvents for effective regeneration or recovery. In the current study, we also investigated the desorption kinetics of verapamil from various Zr-based materials in acetonitrile. The experiments were carried out by adding 0.4 g of verapamil-adsorbed ZrO_2_, Zr-FUM, UiO-66(Zr), UiO-66(Zr)-NH_2_ and UiO-66(Zr)-2COOH into 10 mL of acetonitrile, respectively. After fixed intervals (0–120 min), the quantities of the desorbed verapamil in acetonitrile were analyzed. Figure 1c shows the time profiles of verapamil desorption from verapamil-adsorbed Zr-based materials. From this figure, the desorbed amount of verapamil presents first, a rapid increasing trend (0–5 min), followed by maintaining a near constant (10–120 min). Among the studied materials, the desorbed amount of verapamil from ZrO_2_ illustrates the maximum value, and the concentration of verapamil reached up to 3.67 ng mL^−1^ after a desorption period of 5 min. Both UiO-66(Zr) and UiO-66(Zr)-NH_2_ demonstrate a comparable performance to desorb verapamil from their surfaces. When UiO-66(Zr)-2COOH was used as an adsorbent, it presented the weakest desorption capability in contrast to other materials. By comparing the adsorption (Figure 1a) and desorption (Figure 1c) processes, it can be seen that the desorption processes of verapamil do not track their adsorption behaviors over Zr-based materials. It could be speculated that this difference is a result of the materials’ varying surface properties. For example, when UiO-66(Zr)-2COOH was employed, it would form a strong acid-base interaction with verapamil, which led to its strong adsorption performance to verapamil (Figure 1a). However, in the desorption period, the adsorbed verapamil is hard to be desorbed from its surface. From the desorption curve, as shown in Figure 1c, we can also observe that after 5 min, the desorption amount of verapamil keeps almost constant for all Zr-based materials, presumably attributable to their irreversible adsorption behaviors. From the above discussion, it can be concluded that more adsorption of verapamil on the materials used may not result in increased desorption. 

### 3.2. Elution Behaviors of Verapamil on Zr-Based Material-Coated Paper Substrates

**Figure 2:**
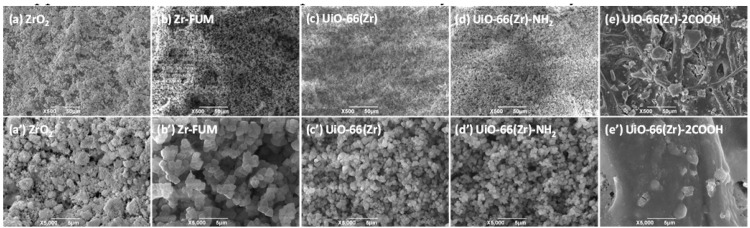
SEM images of (**a**) and (**a’**) ZrO_2_-coated paper, (**b**) and (**b’**) Zr-FUM-coated paper, (**c**) and (**c’**) UiO-66(Zr)-coated paper, (**d**) and (**d’**) UiO-66(Zr)-NH_2_-coated paper, and (**e**) and (**e’**) UiO-66(Zr)-2COOH-coated paper, and (a’–e’) are the close-up images of the representative areas in (a–e). (*Note*: The coated amount of ZrO_2_ and MOFs was 0.5 g, and the solution volume for coating was 100 mL with 0.1 g corn.

As discussed above, one of the major goals of developing MOFs is to eventually allow them to be applied in diverse fields such as separation and analysis. In this study, we coated those materials at the surface of filter paper via a facile vacuum filtration approach [55,56], and then used the obtained papers as substrates for studying the desorption behaviors of different therapeutic drugs and protein samples during paper spray mass spectrometry [53]. Figure 2 shows the typical scanning electron microscope (SEM) images of the obtained paper substrates coated with different Zr-based materials. As displayed in this figure, when blank filter papers were coated with ZrO_2_, Zr-FUM, UiO-66(Zr) and UiO-66(Zr)-NH_2_ (Figure 2a–d,a’–d’), the paper substrates with uniform surface structures were obtained. From their corresponding magnified views, it is obvious that the surfaces of the paper substrates are covered completely by the coated particles with diameters of approximately 0.9–5.4 μm, which is similar to the coated papers with SiO_2_, ZrO_2_, MgO and other MOFs in our recent studies [54,55,56,58]. However, when UiO-66(Zr)-2COOH was employed for coating (Figure 2e,e’), the surface of the achieved paper differed significantly from those mentioned above. The coated paper has a similar cellulosic framework as that of an uncoated one [55], but the pores and its surface are filled with gel particles. This phenomenon could be due to the unstable properties of UiO-66(Zr)-2COOH in the procedures for coating, leading to its dissolution into the solvent used but maintaining its component (Figure S3). From the above results, it can be understood that with a variation in the types of Zr-based materials, the surface structures of the obtained paper substrates changed significantly, which might greatly affect their performances.

**Figure 3:**
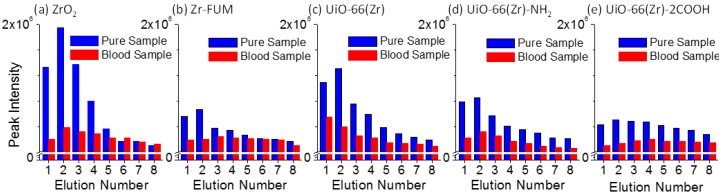
Comparison of the elution behaviors of verapamil samples (Pure Sample: 2 μL of 1:1 methanol/water with 1.0 μg mL^−1^ of verapamil; Blood Sample: 2 μL of blood sample with 1.0 μg mL^−1^ of verapamil) deposited onto the surfaces of various Zr-based material-coated papers: (**a**) ZrO_2_-coated paper, (**b**) Zr-FUM-coated paper, (**c**) UiO-66(Zr)-coated paper, (**d**) UiO-66(Zr)-NH_2_-coated paper, and (**e**) UiO-66(Zr)-2COOH-coated paper (*Note*: The experiments were carried out after the paper had dried. Solvent: 25 μL acetonitrile for each run; Voltage: 3.5 kV).

Paper spray [53,59] is a recently developed ambient ionization source, and has indicated a promising future for direct MS analysis with high quantitation performance owing to its highly simplified procedures and ultra-small amounts of sample consumption. In a paper spray event, the elution or desorption efficiency of target analytes from paper substrate has a pronounced effect on the performance of the final analysis as well as on electrospray efficiency, at the tip of the paper triangle. The elution efficiency is heavily dependent on the interactions between a paper substrate and analytes. Namely, a stronger interaction between them would lead to a weaker elution efficiency at a given experimental condition. To get a better understanding of the interactions between Zr-based material-coated papers and a typical therapeutic drug verapamil, the elution efficiency of two series of verapamil samples, including pure verapamil sample spots and dried blood spots containing verapamil, was investigated intensively. The verapamil sample spots were prepared by dropping 2 μL of either 1:1 methanol/water solution (labeled as the pure sample in Figure 3) or blood sample containing verapamil (1.0 μg mL^−1^) (labeled as the blood sample) onto the paper and the substrate was dried completely. The detailed experimental procedure was similar to our previous report [60]. Namely, solvent was added multiple times to produce several paper spray events using the same substrate, bearing a single sample spot, while the signal of fragment ion *m*/*z* 303 from verapamil was monitored. A spray solvent of 25 μL of acetonitrile was used each time, and the solvent was not added until the monitored ion signal decreased to a minimum and the spray solvent was exhausted. Figure 3 shows the elution efficiency of the verapamil samples deposited onto the surfaces of various Zr-based material-coated papers. For the pure verapamil sample, a much higher peak intensity was observed than that from the blood sample containing verapamil, and the former was approximately 2.0–6.5-fold higher than the latter in the first elution event. This fact could be owing to the effect of the blood matrix on the elution efficiency of verapamil during paper spray. In addition, the peak intensity of verapamil demonstrates first an increasing trend followed by a decrease, with an increased elution number except for UiO-66(Zr)-coated paper in the blood sample analysis. In the second or third spray event, the elution efficiency presents the highest value. Also, it can be seen that ZrO_2_ resulted in the highest elution efficiency for the pure sample (1:1 methanol/water solution containing verapamil) followed by UiO-66(Zr) and UiO-66(Zr)-NH_2_. When Zr-FUM and UiO-66(Zr)-2COOH were used, the elution efficiency was weakest. By comparing the elution efficiency of verapamil from different Zr-based material-coated papers (Figure 3) with their desorption performances in solution systems (Figure 1c), both present a similar trend, suggesting that the elution behavior of verapamil from studied materials during the paper spray process is similar to its desorption in a solution system. As the studied drug verapamil was spiked into the blood sample for the paper spray analysis (Figure 3), the performances of ZrO_2_ and UiO-66(Zr) switched, at which time the elution efficiency of verapamil from the latter was 2.7-fold better than that from the former in the first paper spray event. Generally, when a complex sample is deposited on a piece of paper substrate for paper spray mass spectrometry, just one spray event is applied. This fact suggests that UiO-66(Zr) is a promising coating material for paper spray in the analysis of various analytes (e.g., therapeutic drugs) due to its special tolerance of a complex sample matrix, such as blood [54]. 

### 3.3. Elution Behaviors of Therapeutic Drugs and Proteins

**Figure 4:**
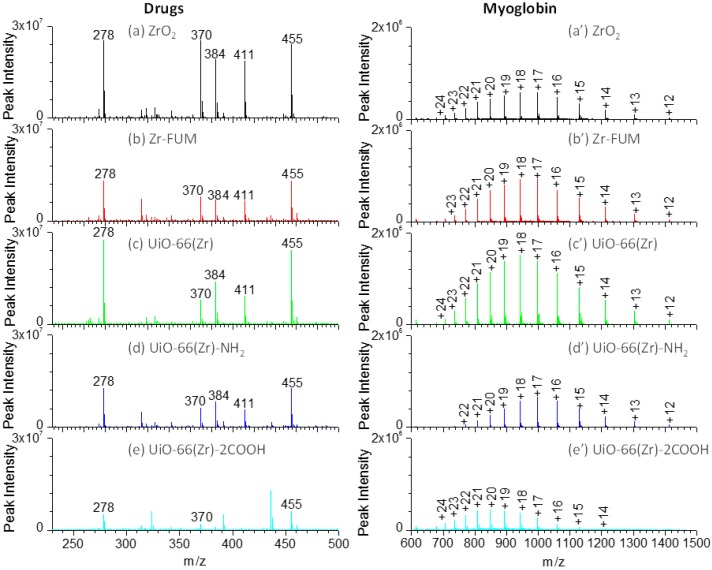
Mass spectra of a drug mixture containing amitriptyline (*m*/*z* 278), amisulpride (*m*/*z* 370), quetiapine (*m*/*z* 384), risperidone (*m*/*z* 411) and verapamil (*m*/*z* 455) (**a–e**) and myoglobin (**a’–e’**) using various Zr-based material-coated papers as substrates for paper spray: (**a**) and (**a’**) ZrO_2_-coated paper, (**b**) and (**b’**) Zr-FUM-coated paper, (**c**) and (**c’**) UiO-66(Zr)-coated paper, (**d**) and (**d’**) UiO-66(Zr)-NH_2_-coated paper, and (**e**) and (**e’**) UiO-66(Zr)-2COOH-coated paper (*Note*: For drug analysis: sample volume, 2 μL of 1:1 methanol/water containing 10 μg mL^−1^ of corresponding drugs; solvent, 25 μL acetonitrile; voltage: 3.5 kV. For myoglobin analysis: sample volume, 5 μL; sample concentration, 50 μg mL^−1^ for myoglobin; solvent: 25 μL 1:1 methanol/water; voltage: 3.5 kV).

The accurate measurement of therapeutic drugs plays a crucial role in drug discovery and disease therapy. Paper spray mass spectrometry has demonstrated its potential in rapid therapeutic drug monitoring [59,60,61]. However, the selection of a paper substrate is critical to the overall capability of the paper spray analysis. To comprehensively compare the performance of the Zr-based material-coated paper substrates in paper spray, several therapeutic drugs, including amitriptyline (*m*/*z* 278), amisulpride (*m*/*z* 370), quetiapine (*m*/*z* 384), risperidone (*m*/*z* 411) and verapamil (*m*/*z* 455) as shown in Figure S1, were analyzed. Figure 4a–e shows the mass spectra of those drugs using the coated papers as substrates for the paper spray. As shown in this figure, when ZrO_2_ and UiO-66(Zr) were used as coating materials, both demonstrated a comparable performance and indicated the highest signals in the analysis of those drugs, although the peak intensity of amisulpride, quetiapine, and risperidone was a little higher on ZrO_2_ than those on UiO-66(Zr), in agreement with the above discussion (Figure 1c and Figure 3). However, the peak intensity of the tested drugs sharply decreased when Zr-FUM and UiO-66(Zr)-NH_2_ were employed as the coating materials, which is around half of those from ZrO_2_ and UiO-66(Zr). As UiO-66(Zr)-2COOH was used, it demonstrated the poorest performance presumably due to the strong acid/base, hydrogen bond and van de Waals interactions between it and the tested basic drugs. 

In the current study, we also explored the capability of various Zr-based material-coated paper substrates in an analysis of proteins during paper spray mass spectrometry. Figure 4a’–e’ shows the mass spectra of a typical protein, myoglobin (Figure S1), using various Zr-based material-coated papers as substrates for paper spray mass spectrometry. For myoglobin, by varying the substrate from ZrO_2_, Zr-FUM to UiO-66(Zr)-coated papers, the most abundant peak occurred at *m*/*z* 942 (+18), but the peak intensity gradually increased, possibly a result of the different surface properties of those materials. When UiO-66(Zr)-NH_2_ and UiO-66(Zr)-2COOH were used as coating materials, relatively weaker signals were obtained, especially for UiO-66(Zr)-2COOH-coated paper. This case could be ascribed to the stronger acid-base or hydrogen bond interactions between myoglobin and the coated materials. The charge state distribution (CSD) of protein ions provides an effective means of monitoring changes in protein conformation induced by changing experimental conditions (e.g., solvent pH, composition and temperature) [57,62,63]. Herein, the surface properties of Zr-based material-coated paper substrates were characterized by an average charge state *z*_ave_ of tested proteins. As displayed in Figure 4a’–e’, when ZrO_2_-, Zr-FUM- and UiO-66(Zr)-coated papers were used for the analysis of myoglobin, the *z*_avg_ values were in the range of 17.93–18.20, suggesting that the surface properties of those materials have little effect on the myoglobin conformation. However, when UiO-66(Zr)-NH_2_-coated paper was used, the *z*_avg_ value shifted to 17.10. In contrast, the value became 19.95 as UiO-66(Zr)-2COOH was employed as a coating material. By comparing the surface structures of UiO-66(Zr)-NH_2_ and UiO-66(Zr)-2COOH, it could be found that when the surface of a coated material displays basic properties, myoglobin is preferable to form lower charge ions, whereas higher charge ions are favorable when coated materials with acidic properties are used. 

## 4. Conclusions

In summary, the adsorption and desorption performances of various Zr-based materials [e.g., Zr-FUM, UiO-66(Zr), UiO-66(Zr)-NH_2_ and UiO-66(Zr)-2COOH] as well as ZrO_2_ have been systematically investigated using paper spray mass spectrometry. The results demonstrated that in contrast to other materials, UiO-66(Zr)-2COOH presented a superior adsorption ability to the studied therapeutic drug verapamil due to their strong acid-base or hydrogen-bond interactions. As those materials were used for desorption experiments, ZrO_2_ showed the most favorable performance. In addition, we coated the investigated Zr-based materials onto the surface of filter paper and studied the elution or desorption behaviors of different drugs and proteins from the coated papers. ZrO_2_-coated paper was found to display the most favorable elution behaviors to studied drugs prepared in pure solvent, whereas UiO-66(Zr)-coated paper demonstrated special tolerance to a sample matrix, with the optimal performance to drugs in a complex matrix such as blood and protein analysis. The present study not only paves the way to explore the adsorption and desorption performances of various Zr-based materials with paper spray mass spectrometry, but also provides a deep insight into the application of metal–organic frameworks in analyses of different samples. 

## Figures and Tables

**Table 1:** Names, structures and texture properties of different metal–organic frameworks MOFs.

Name	ZrO_2_	Zr-FUM	UiO-66(Zr)	UiO-66(Zr)-NH_2_	UiO-66(Zr)-2COOH
Structure	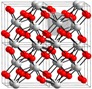	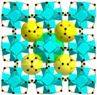	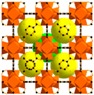	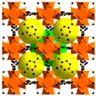	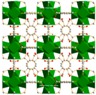
BET surface area *^a^* (m^2^ g^−1^)	19.7	735.2	1204.2	1070.1	450.3
Pore volume *^b^* (cm^3^ g^−1^)	0.177	0.342	0.590	0.465	0.245
Average pore size (nm)	28.4	0.72	2.36	1.89	3.81

*^a^* Using the standard Brunauer–Emmett–Teller (BET) method; *^b^* Using the Barrett–Joyner–Halenda (BJH) method.

**Table 2:** Adsorption kinetic parameters for the adsorption of verapamil on various Zr-based MOFs in 1.0 μg mL^−1^ verapamil solution using acetonitrile as a solvent.

MOFs	Pseudo-First-Order Model				Pseudo-Second-Order Model			
	Experimental *q*_e_ (mg g^−1^)	Calculated *q*_e_ (mg g^−1^)	*k*_1_ (g mg^−1^ min^−1^)	R^2^	Experimental *q*_e_ (mg g^−1^)	Calculated *q*_e_ (mg g^−1^)	*k*_2_ (g mg^−1^ min^−1^)	R^2^
ZrO_2_	0.0170	0.0127	0.0295	0.9666	0.0170	0.0174	7.407	0.9925
Zr-FUN	0.0141	0.0081	0.0263	0.9674	0.0141	0.0140	15.893	0.9920
UiO-66(Zr)	0.0157	0.0118	0.0318	0.9724	0.0157	0.0162	8.235	0.9939
UiO-66(Zr)-NH_2_	0.0180	0.0094	0.0290	0.9485	0.0180	0.0179	16.142	0.9953
UiO-66(Zr)-2COOH	0.0211	0.0084	0.0272	0.9301	0.0211	0.0209	19.159	0.9967

## Supplementary Materials

The following materials are available at www.mdpi.com/1996-1944/10/7/769/s1. Figure S1: Structures of verapamil, risperidone, amitriptyline, amisulpride, quetiapine and myoglobin; Figure S2: XRD patterns of different Zr-based materials: (a) ZrO2, (b) Zr-FUM, (c) UiO-66(Zr), (d) UiO-66(Zr)-NH2 and (e) UiO-66(Zr)-2COOH; Figure S3: FT-IR spectra of (a) the collected product after sonicating UiO-66(Zr)-2COOH in aqueous solution for 20 min and (b) intact UiO-66(Zr)-2COOH.
