# Supplementary material for: Study of Adsorption and Desorption Performances of Zr-Based Metal–Organic Frameworks Using Paper Spray Mass Spectrometry
Source: Materials (Basel). 2017 Jul 8;10(7):769. doi: 10.3390/ma10070769 (PMC5551812; doi:10.3390/ma10070769)
Supplement: Supplementary file 1 [file materials-10-00769-s001.pdf]

Supplementary Material

Study of Adsorption and Desorption Performances of Zr-Based Metal–Organic Frameworks Using Paper Spray Mass Spectrometry

Xiaoting Wang [1], Ying Chen [2], Yajun Zheng [1] and Zhiping Zhang [1,*]

[1] School of Chemistry and Chemical Engineering, Xi'an Shiyou University, Xi'an 710065, China; 18629343797@163.com (X.W.); returnshiyou@xsyu.edu.cn (Y.Z.)
[2] Clinical Analysis Laboratory, Xi'an Mental Health Center, Xi'an 710061, China; 292488303@ qq.com
* Correspondence: zhangzp0304@gmail.com; Tel.: +86-29-8838-2694

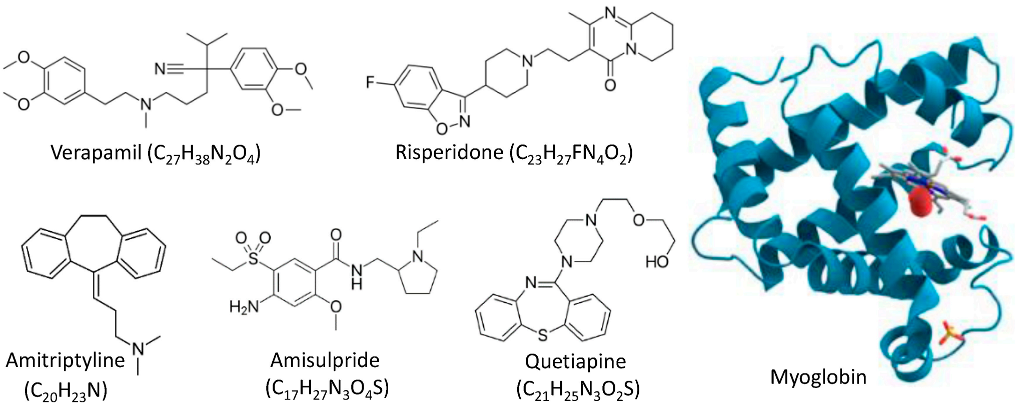

Figure S1. Structures of verapamil, risperidone, amitriptyline, amisulpride, quetiapine and myoglobin.

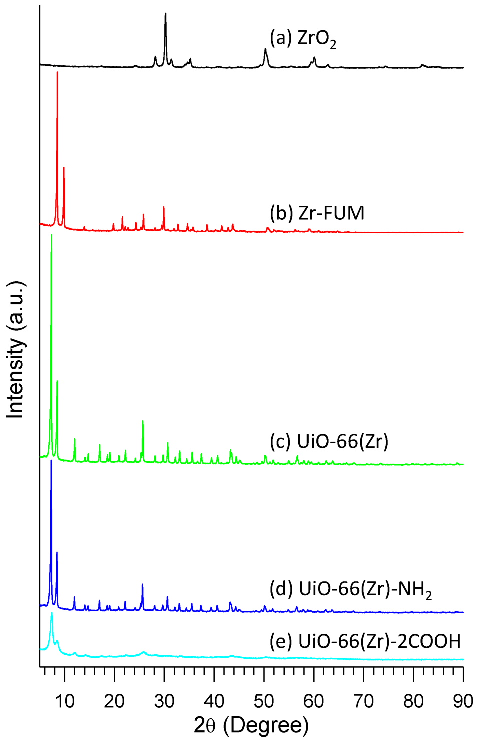

Figure S2. XRD patterns of different Zr-based materials: (**a**) ZrO_2, (**b**) Zr-FUM, (**c**) UiO- 66(Zr), (**d**) UiO-66(Zr)-NH_2 and (**e**) UiO-66(Zr)-2COOH.

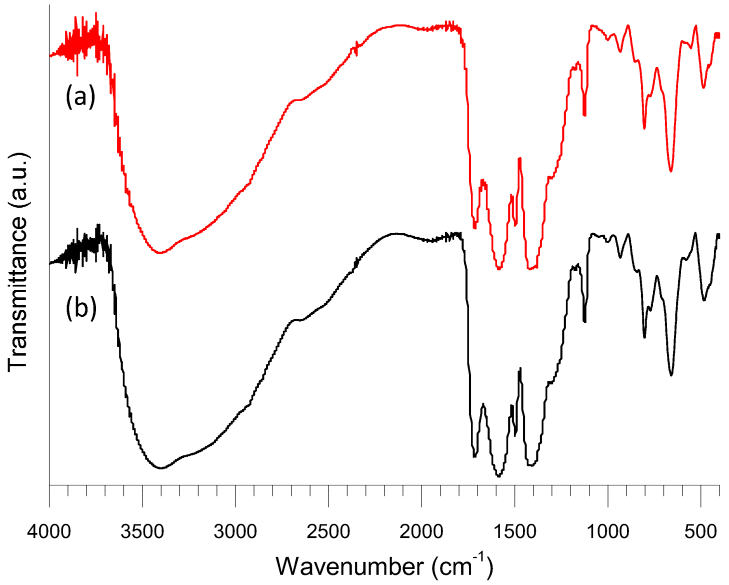

Figure S3. FT-IR spectra of (**a**) the collected product after sonicating UiO-66(Zr)-2COOH in aqueous solution for 20 min and (**b**) intact UiO-66(Zr)-2COOH.

© 2017 by the authors. Submitted for possible open access publication under the terms and conditions of the Creative Commons Attribution (CC BY) license (http://creativecommons.org/licenses/by/4.0/).
